# Diagnostic performance of convolutional neural networks for dental sexual dimorphism

**DOI:** 10.1038/s41598-022-21294-1

**Published:** 2022-10-14

**Authors:** Ademir Franco, Lucas Porto, Dennis Heng, Jared Murray, Anna Lygate, Raquel Franco, Juliano Bueno, Marilia Sobania, Márcio M. Costa, Luiz R. Paranhos, Scheila Manica, André Abade

**Affiliations:** 1 Centre of Forensic and Legal Medicine and Dentistry, University of Dundee, Dundee, UK; 2 Department of Therapeutic Stomatology, Institute of Dentistry, Sechenov University, Moscow, Russia; 3 Computer Vision Solutions, Rumina S.A, Belo Horizonte, Minas Gerais, Brazil; 4 Department of Preventive and Social Dentistry, Federal University of Uberlandia, Av. Pará 1720, Bloco 2G, Sala 1, Campus Umuarama, Uberlândia, Minas Gerais Brazil; 5 Division of Oral Radiology, Faculdade Sao Leopoldo Mandic, Campinas, Brazil; 6 Division of Forensic Dentistry, Faculdade Sao Leopoldo Mandic, Campinas, Brazil; 7 Department of Removable Prosthodontics, Federal University of Uberlandia, Uberlândia, Brazil; 8 Computer Science, Federal Institute of Science and Technology, Barra do Garças, Brazil

**Keywords:** Dental anthropology, Dental radiology, Forensic dentistry

## Abstract

Convolutional neural networks (CNN) led to important solutions in the field of Computer Vision. More recently, forensic sciences benefited from the resources of artificial intelligence, especially in procedures that normally require operator-dependent steps. Forensic tools for sexual dimorphism based on morphological dental traits are available but have limited performance. This study aimed to test the application of a machine learning setup to distinguish females and males using dentomaxillofacial features from a radiographic dataset. The sample consisted of panoramic radiographs (*n* = 4003) of individuals in the age interval of 6 and 22.9 years. Image annotation was performed with V7 software (V7labs, London, UK). From Scratch (FS) and Transfer Learning (TL) CNN architectures were compared, and diagnostic accuracy tests were used. TL (82%) performed better than FS (71%). The correct classifications of females and males aged ≥ 15 years were 87% and 84%, respectively. For females and males < 15 years, the correct classifications were 80% and 83%, respectively. The Area Under the Curve (AUC) from Receiver-operating Characteristic (ROC) curves showed high classification accuracy between 0.87 and 0.91. The radio-diagnostic use of CNN for sexual dimorphism showed positive outcomes and promising forensic applications to the field of dental human identification.

## Introduction

Several techniques used in forensic sciences rely on subjective operator-dependent procedures^1^. The decision-making process behind these procedures requires experience and may lead to error rates with a significant impact in practice^2^. Important contributions of forensic dentistry to forensic sciences emerged from radio-diagnostic procedures, such as dental charting for human identification^3–5^, and dental staging for age estimation^6–10^. Computer-based tools were developed to create a man–machine interface and reduce bias from the operator’s side. Software like KMD PlassData DVI™ (KMD s/a, Ballerup, Denmark) added quality control procedures to the reconciliation process, made disaster victim identification less time-consuming, and guaranteed more straightforward human identifications^11^. In dental age estimation, promising automated techniques abbreviated the number of manual interactions needed to allocate developmental stages to teeth examined on radiographs^12^. While dental charting has a fundamental role in comparative human identification, dental age estimation contributes indirectly as a reconstructive factor.

Among the reconstructive factors, sex plays a fundamental part in narrowing lists of missing persons^13^. When biological/physical sex-related parameters are available they may lead to binary segregation of the victims (into males and females) and limit the number of required antemortem (AM) and postmortem (PM) comparisons^14^. A recent systematic literature review with over a hundred eligible studies highlighted the importance of dentomaxillofacial features in the process of sexual dimorphism^15^. According to the authors, the existing techniques for sexual dimorphism based on teeth can be biochemical (e.g. from the analysis of dental tissues), metric (namely measuring teeth), and non-metric (e.g. relying on dental morphology)^15^. Biochemical techniques seem to be more accurate^15^ and represent the current state-of-the-art when it comes to dental analyses. However, the application of these techniques in practice is restricted because they require advanced facilities and tools that are not usually available in most medicolegal institutes, especially in developing countries.

The most common techniques debated in the current scientific literature fall within the group of metric analyses, in which linear measurements (mesiodistal width and intercanine distance) and volumetric assessments can be performed ex-vivo or through 2D (radiographic/photographic) 3D (tomographic scan) imaging^16^. In this context, examiner reproducibility is a drawback since millimetric measurements and volumetric analyses require extensive calibration and training. In order to reduce operator-dependent interactions, artificial intelligence could figure as an option to enhance diagnostic performances of sex estimation techniques. Machine learning algorithms are known to learn underlying relationships in data and support the decision-making process (or even make decisions without requiring explicit instructions)^17^. In 1989, the concept of a Convolutional Neural Network (CNN) was introduced and demonstrated enormous potential for tasks related to computer vision. CNNs are among the best learning algorithms for understanding images and have demonstrated exemplary performance in tasks related to image segmentation, classification, detection, and retrieval^18^. One of the most outstanding features of CNNs is their ability to explore spatial or temporal correlation in the data. The CNN topology is divided into several learning stages that consist of a combination of convolutional layers, non-linear processing units, and subsampling layers^19^. Since the late ’90 s, several improvements in the learning architecture of CNNs were made to enable the assessment of large, heterogeneous, complex, and multiclass datasets^19^. The proposed innovations included the modification of image processing units, optimization for the assessment of parameters and hyperparameters, new “design” patterns, and layer connectivity ^18,20,21^.

In this scenario, artificial intelligence could find productive grounds for the use of radiographic datasets and could be challenged for sexual dimorphism. However, given the existing scientific literature and the morphological parameters currently known to be dimorphic (e.g. the maxillary sinuses^22^), testing the performance of machine learning algorithms to estimate the sex of adults would be merely confirmatory. In order to propose a real challenge to artificial intelligence, sexual dimorphism could be performed with a sample of children and juveniles—a population in which anthropological indicators of sex are not well-pronounced or at least not fully expressed.

In country-specific jurisdictions, the admissibility of evidence in Court depends on several technical aspects, including the knowledge about the error of the method (factor including in Daubert’s rule, for instance). With that in mind, testing forensic solutions developed with artificial intelligence, and investigating the accuracy of the method (and inherent error) are initial steps prior to implementing computer-aided tools in practice. This diagnostic study aimed to use a radiographic dataset in a machine learning setup to promote an automated process of sexual dimorphism based on dentomaxillofacial features of children and juveniles.

## Materials and methods

### Ethical aspects and study design

This was a diagnostic study with retrospective sample collection. The methodological architecture was based on a medical imaging dataset to feed machine learning within the context of artificial intelligence. Informed consent was waived because the study was observation and required retrospective sampling from a pre-existing image database, but ethical approval was obtained from the Ethics Committee in Human Research of Faculdade Sao Leopoldo Mandic. The Declaration of Helsinki (DoH), 2013, was followed to assure ethical standards in this medical research. The sample was collected from a pre-existing institutional image database. Hence, no patient was prospectively exposed to ionizing radiation merely for research purposes. All the images that populated the database were obtained for diagnostic, therapeutic, or follow-up reasons.

### Sample and participants

The sample consisted of panoramic radiographs (*n* = 4003; 1809 males and 2194 females) collected according to the following eligibility criteria: Inclusion criteria—radiographs of male and female Brazilian individuals with age between 6 and 22.9 years. Exclusion criteria—panoramic radiographs missing patient’s information about sex, date of birth, and date of image acquisition; visible bone lesions and anatomic deformity; the presence of implants and extensive restorative materials; severely displaced and/or supernumerary teeth. The radiographs were obtained from a private oral imaging company in the Central-Western region of Brazil. The images were imported to an Elitebook 15.6" FHD Laptop with i5 (Hewlett-Packard, Palo Alto, CA, USA) for analysis.

The annotations were accomplished by three trained observers, with experience in forensic odontology, supervised by a forensic odontologist with 11 years of practice in the field. A bounding-box tool was used to annotate the region of interest in Darwin V7 (V7 Labs, London, UK) software package^23^. Vertically (y-axis), the box was positioned covering the apical region of the most superior teeth whilst the lower limit covered the apical region of the most inferior teeth. Laterally (x-axis), the box ended right after the third molars, bilaterally. The final selection of the region of interest was represented by a rectangular box covering all the teeth visible in the panoramic radiograph. The images were anonymized for annotation, hiding age and sex information. The software registered the annotations that were later tested for association with sex.

### Pre-processing and training approach

**Figure 1:**
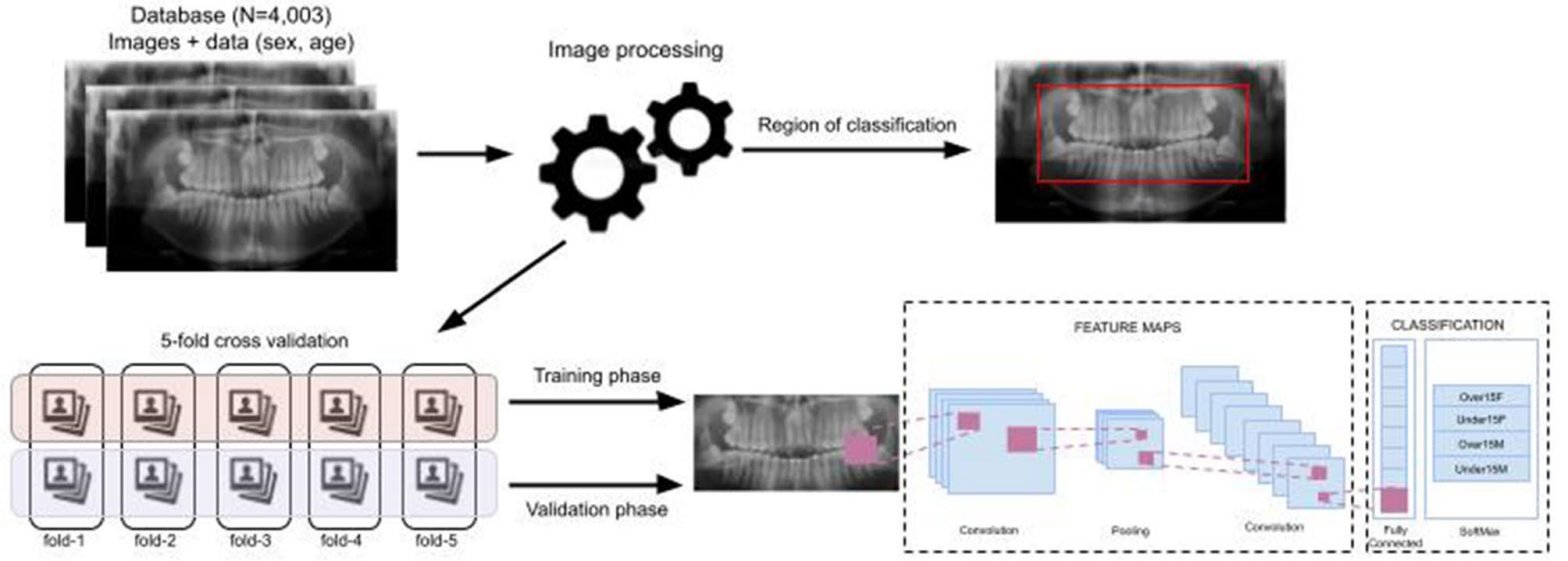
Model structured for this study showing the workflow from sampling, image processing, annotation, cross-validation, training/validation to classification.

The full dataset of panoramic radiographs was initially divided into the age groups “under 15 years" (*n* = 2,254) and “equal or older 15 years" (*n* = 1,749). This division was justified to challenge the network regarding the sexual dimorphism. In children, sexual dimorphism is more difficult because the expression of external sexual features is not pronounced. Hence, the age of 15 years represents a transitional point to a fully developed permanent dentition (except for the third molars)^8^. Normally, all the permanent teeth will have fully developed crowns around this age^8^. The roots, if not developed, will present a late stage of formation^16^. In each age group (< 15 years vs. ≥ 15 years) a single problem was established: sexual dimorphism, and a binary outcome was expected regarding sex (male *vs.* female), and age (< 15 years vs. ≥ 15 years). Hence, four classes were considered in this study: under 15 males vs. under 15 females; and over 15 males vs. over 15 females (Fig. 1).

Next, the images were pre-processed preserving high-level of detail and signal-to-noise ratio while avoiding photometric nonlinearity and geometric distortion. Initially, in this study, we used eight CNNs architectures namely DenseNet121, InceptionV3, Xception, InceptionResNetV2, ResNet50, ResNet101, MobileNetV2, and VGG16. DenseNet121 was selected in this study because this is one of the most successful models of recent times, and is available from open sources (e.g. Pytorch, TensorFlow and Keras API). Additionally, it must be noted that DenseNet121 outperformed the other architectures during a pilot study that we performed with 100 epochs (Table 1). Table 2 shows the characteristics of the architecture models used in this study.

**Table 1:** Summarized results of the metrics of the seven models evaluated in a pilot test to support the decision-making process for the selection of a network.

CNN model	Architecture	K-fold 5	Loss	Metrics				
				Accuracy	F_1_-score	Precision	Recall	Specificity
DenseNet121 100 epochs Batch size=32	TL	Fold 1	0.7780	0.8327	0.8193	0.8203	0.8185	0.9213
		Fold 2	0.6892	0.8227	0.7920	0.7920	0.7920	0.9112
		Fold 3	0.6635	0.8114	0.7804	0.7808	0.7800	0.9121
		Fold 4	0.7392	0.8162	0.8159	0.8169	0.8149	0.9320
		Fold 5	0.6757	0.8262	0.8242	0.8261	0.8224	0.9334
		Average	0.7091	0.8218	0.8064	0.8072	0.8056	0.9220
InceptionV3 100 epochs Batch size=16	TL	Fold 1	0.8517	0.7640	0.7608	0.7649	0.7573	0.9037
		Fold 2	0.5928	0.7640	0.7564	0.7615	0.7524	0.8953
		Fold 3	0.7088	0.7503	0.7437	0.7464	0.7414	0.8988
		Fold 4	0.6979	0.7712	0.7673	0.7715	0.7637	0.9095
		Fold 5	0.6236	0.7599	0.7588	0.7679	0.7512	0.9043
		Average	0.6950	0.7619	0.7574	0.7625	0.7532	0.9023
Xception 100 epochs Batch size=32	TL	Fold 1	0.9429	0.7852	0.7749	0.7758	0.7740	0.9084
		Fold 2	0.7903	0.8039	0.7732	0.7736	0.7728	0.9071
		Fold 3	1.0323	0.7702	0.7603	0.7610	0.7596	0.9034
		Fold 4	0.8688	0.8087	0.8079	0.8083	0.8075	0.9312
		Fold 5	0.9424	0.7875	0.7871	0.7882	0.7862	0.9233
		Average	0.9154	0.7911	0.7807	0.7814	0.7800	0.9147
InceptionResNetV2 100 epochs Batch size=32	TL	Fold 1	0.9598	0.7915	0.7618	0.7629	0.7608	0.9053
		Fold 2	0.9619	0.8127	0.8007	0.8024	0.7992	0.9142
		Fold 3	0.9329	0.8064	0.7950	0.7955	0.7944	0.9132
		Fold 4	0.8800	0.7962	0.7965	0.7968	0.7962	0.9272
		Fold 5	0.7088	0.8324	0.8324	0.8336	0.8312	0.9387
		Average	0.8886	0.8078	0.7973	0.7982	0.7964	0.9197

CNN model	Architecture	K-fold 5	Loss	Metrics				
				Accuracy	F_1_-score	Precision	Recall	Specificity
ResNet50 100 epochs Batch size=32	TL	Fold 1	0.9303	0.7915	0.7626	0.7645	0.7608	0.9041
		Fold 2	1.0381	0.8002	0.7881	0.7903	0.7860	0.9118
		Fold 3	0.8592	0.8177	0.7872	0.7872	0.7872	0.9109
		Fold 4	0.9334	0.8062	0.8066	0.8071	0.8062	0.9297
		Fold 5	0.7910	0.8062	0.8072	0.8082	0.8062	0.9304
		Average	0.9104	0.8043	0.7903	0.7915	0.7893	0.9174
ResNet101 100 epochs Batch size=32	TL	Fold 1	0.9598	0.8014	0.7712	0.7721	0.7704	0.9064
		Fold 2	0.8728	0.8102	0.7977	0.7987	0.7968	0.9175
		Fold 3	0.9338	0.7952	0.7819	0.7827	0.7812	0.9110
		Fold 4	0.8091	0.7962	0.7968	0.7989	0.7950	0.9229
		Fold 5	0.8373	0.8075	0.8064	0.8067	0.8062	0.9308
		Average	0.8826	0.8021	0.7908	0.7918	0.7899	0.9177
MobileNetV2 100 epochs Batch size=32	TL	Fold 1	0.7950	0.7990	0.7682	0.7710	0.7656	0.9043
		Fold 2	1.0042	0.7777	0.7501	0.7516	0.7487	0.8989
		Fold 3	1.0015	0.7865	0.7752	0.7752	0.7752	0.9075
		Fold 4	0.8395	0.7837	0.7838	0.7838	0.7837	0.9228
		Fold 5	0.6802	0.8075	0.8086	0.8098	0.8075	0.9248
		Average	0.8641	0.7909	0.7772	0.7783	0.7761	0.9117
VGG16 100 epochs Batch size=32	TL	Fold 1	0.6843	0.8064	0.7769	0.7775	0.7764	0.9071
		Fold 2	0.6431	0.8439	0.8125	0.8125	0.8125	0.9197
		Fold 3	0.5552	0.8064	0.7949	0.7954	0.7944	0.9105
		Fold 4	0.5840	0.7362	0.7376	0.7509	0.7262	0.8727
		Fold 5	0.6014	0.7024	0.6990	0.7064	0.6924	0.8725
		Average	0.6136	0.7791	0.7642	0.7685	0.7604	0.8965

*CNN* convolutional neural network using transfer-learning architecture.

**Table 2:** Specifics of the CNN architectures applied and tested in this study.

Model	Size (MB)	Parameters (M)	Depth	Image size	Hyperparameters				
					Optimization algorithm	Batch size	Momentum	Weight decay	Learning rate
DenseNet121	33	8.1	121	224 × 224	SGD	32	0.9	1e-4 ~ 1e-6	Base Ir = 0.001 Max Ir = 0.00006 Step size = 100 Mode: triangular
ResNet50	98	25.6	107	224 × 224					
ResNet101	171	44.7	209	224 × 224					
Xception	88	22.9	81	299 × 299					
InceptionV3	92	23.9	189	299 × 299					
InceptionResNetV2	215	55.9	449	299 × 299					
VGG16	526	138.4	16	224 × 224					
MobileNetV2	14	3.5	105	224 × 224					

*CNN* Convolutional Neural Network, *MB* MegaBytes, *M* Million Parameters, *SGD* Stochastic Gradient Descent.

In this study, we evaluated the DenseNet121 architecture using two training approaches: From Scratch (FS) and Transfer Learning (TL). With FS the network weights are not inherited from a previous model but are randomly initialized. It requires 1) a larger training set, 2) the risk of overfitting`1`^28^ is higher since the network has no experience from previous training sessions, and 3) the network needs to rely on the input data to define all inherent weights. However, this approach allows the creation of a network topology that can work towards a specific problem/question. TL is a method that reuses models applied to specific tasks as a starting point for new domains of interest. Consequently, the network borrows data (with original labels) or extracts knowledge from related fields to obtain the highest possible performance in the area of interest^24,25^. As per standard practices, TL can be applied using a base neural network as a fixed feature extractor. This way the images of the target dataset are fed to the deep neural network. Later, the features that are generated as input to the final layer classifier are extracted^26^. Through these features, a new classifier is built, and the model is created. Specifically for the base network (last layer), a fine-tuning strategy is added, and the weights of previous layers are also modified. We used pre-trained weights based on the ImageNet model^27^ and implemented transfer learning to best fit our dataset.

To avoid overfitting and improve the generalizability of the evaluated models (due to the quantitative restriction of images in the data set) we used a computational framework (Keras^29^) for pre-processing layers to create a pipeline augmentation layers of image data—which can be used as an independent pre-processing code in non-Keras^30^ workflows. These layers apply random augmentation transformations to a batch of images and are only active during training^30^. Table 3 presents each layer with its respective implemented parameters.

**Table 3:** Image data augmentation layers and parameters.

Layer	Parameter
RandomTranslation	height_factor = 0.1, width_factor = 0.1, fill_mode = ’reflect’
RandomFlip	mode = ’horizontal_and_vertical’
RandomRotation	factor = 0.1, fill_mode = ’reflect’, interpolation = ’bilinear’
RandomContrast	factor = 0.1

A stochastic optimization algorithm (SGD) was used to optimize the training process. We initially set a base learning rate of 1 × 10^−3^. The base learning rate was decreased to 6 × 10^−6^ with increased iterations. In the validation process, we used the k-fold cross-validation method^31,32^. The dataset was divided into 5 (k) mutually exclusive subsets of the same size (five sets of 20% of the sample). This strategy creates a subset (20%) to be used for the tests and the remaining k − 1 (80%) is used to estimate the parameters (training). The five sets were dynamic over five repetitions for each of the architectures (TL and FS). It means that all the training samples had a different (randomly selected) dataset built from the original sample. Hence, images used during the training process were not used in the subsequent validation stage within the same k-fold training-test. After this process quantification of the model accuracy is feasible.

### Diagnostic metrics

To evaluate the (radio-diagnostic) classification performance of the proposed architecture, the loss, overall accuracy, F1-scores, precision, recall, and specificity were selected as the accuracy performance metrics (Table 4). In the training stage, the internal weights of the model are updated during several iterations. We supervised each iteration in the training period, registering the weights with the best predictive power of the model determined by the overall accuracy metric.

**Table 4:** Diagnostic metrics used to evaluate the performance of the investigated CNN architectures.

Metrics	Description
Loss	A loss function indicates how well the model assimilates the dataset. The loss function will output a higher value if the predictions are off the actual target. Since our problem/question relies on a multi-class classification, we used cross-entropy within our loss function
Accuracy	The accuracy of a machine learning classification algorithm is one way to measure how often the algorithm classifies a data point correctly. This can be understood as the number of items correctly identified as either true positive or true negative out of the total number of items
F_1_-score	Represents the average of precision and recall and measures the effectiveness of identification when recall and precision have balanced importance
Precision	Agreement of true class labels with machine’s predictions. It is calculated by summing all true positives and false positives in the system, across all classes
Recall	Effectiveness of a classifier to identify class labels. It is calculated by summing all true positives and false negatives in the system, across all classes
Specificity	Known as the true negative rate. This function calculates the proportion of actual negative cases that have gotten predicted as negative by our model

*CNN* convolutional neural network.

Additionally, this study quantified the performance of the CNN into a confusion matrix^33^ for FS and TL. The matrix contains information about true (real) and predicted classifications accomplished the CNN. This approach helps on finding and reducing bias and variance issues and enables adjustments capable of producing more accurate results. Another approach used in this study was the Receiver Operating Characteristic (ROC) curve^34^, which is a diagnostic tool to enable the analysis of classification performances represented by sensitivity, specificity, and area under the curve (AUC). Visual outcomes were illustrated with gradient-weighted class activation mapping (Grad-CAM) to indicate the region on the panoramic radiograph that was more activated during the machine-guided decision to classify females and males. The study was performed with a Linux machine, with Ubuntu 20.04, an Intel® Core(TM) i7-6800 K processor, 2 Nvidia® GTX Titan Xp 12 GB GPUs, and 64 GB of DDR4 RAM. All models were developed using TensorFlow API version 2.5^35^ and Keras version 2.5 ^29^. Python 3.8.10 was used for algorithm implementation and data wrangling^36^.

## Results

The performance of DenseNet121 architecture tested with FS and TL approaches showed that the former had an overall accuracy rate of 0.71 with a specificity rate of 0.87. With TL, the overall accuracy increased to 0.82 with a specificity rate of 0.92—between K-folds 1–5 TL accuracy floated between 0.81 to 0.83. All the other metrics quantified in this study confirmed the superior performance of TL over FS (Table 5).

**Table 5:** Quantified performances of DenseNet121 with FS and TL architectures.

CNN model	Architecture	K-fold 5	Metrics					
			Loss	Accuracy	F_1_-score	Precision	Recall	Specificity
DenseNet121 100 epochs Batch size = 32	FS	Fold 1	0.6835	0.7215	0.7104	0.7272	0.6959	0.8705
		Fold 2	0.6175	0.7166	0.6863	0.6916	0.6814	0.8627
		Fold 3	0.6203	0.7141	0.7093	0.7133	0.7055	0.8719
		Fold 4	0.6174	0.7200	0.7200	0.7284	0.7124	0.8840
		Fold 5	0.7234	0.7099	0.7061	0.7187	0.6949	0.8844
		Average	0.6524	0.7164	0.7064	0.7159	0.6980	0.8747
	TL	Fold 1	0.7780	0.8327	0.8193	0.8203	0.8185	0.9213
		Fold 2	0.6892	0.8227	0.7920	0.7920	0.7920	0.9112
		Fold 3	0.6635	0.8114	0.7804	0.7808	0.7800	0.9121
		Fold 4	0.7392	0.8162	0.8159	0.8169	0.8149	0.9320
		Fold 5	0.6757	0.8262	0.8242	0.8261	0.8224	0.9334
		Average	0.7091	0.8218	0.8064	0.8072	0.8056	0.9220

*FS* from scratch, *TL* transfer learning.

**Figure 2:**
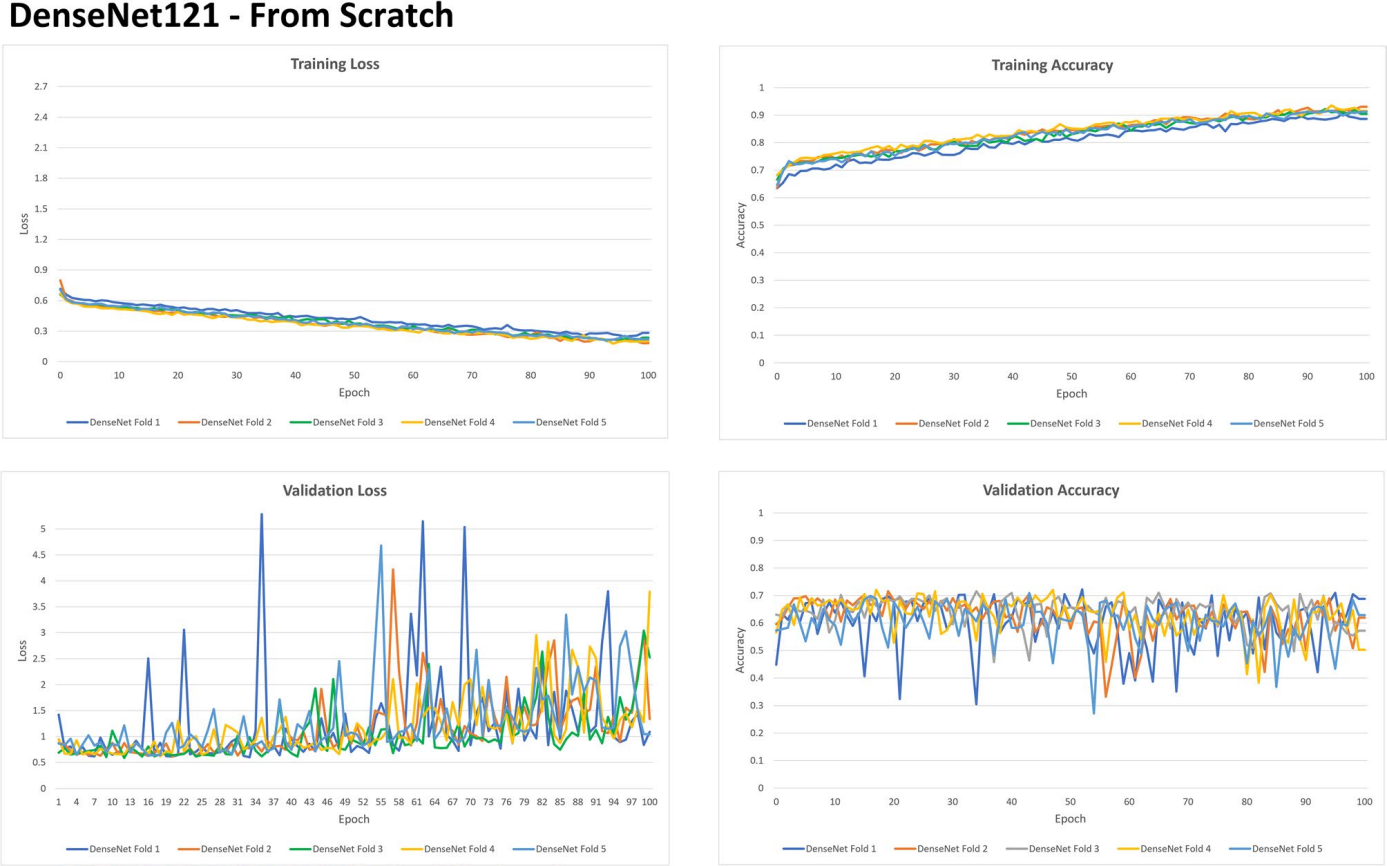
Graphs representing the loss and evolutionary accuracy of the training process and learning validation with From Scratch (FS) architecture in DenseNet121.

**Figure 3:**
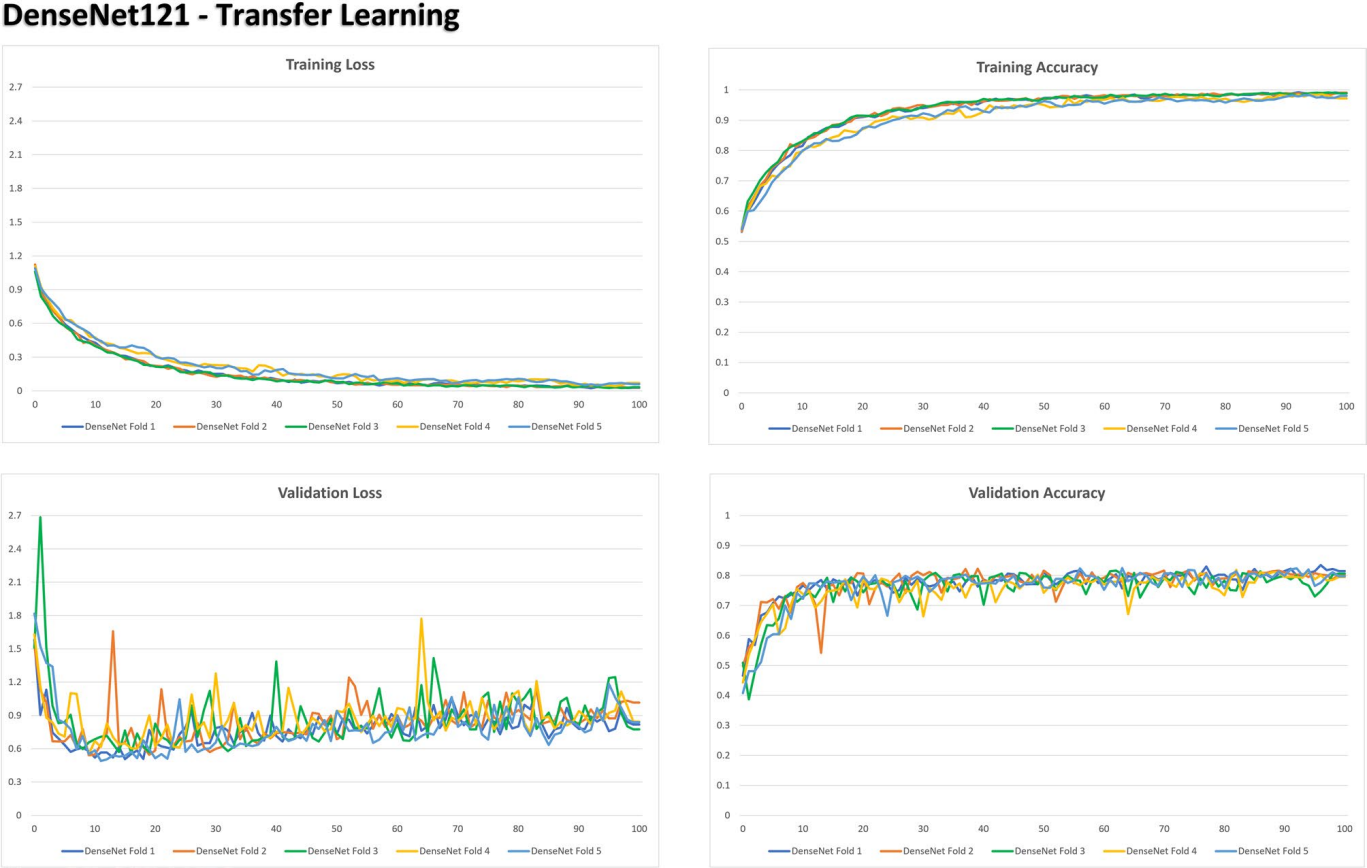
Graphs representing the loss and evolutionary accuracy of the training process and learning validation with Transfer Learning (TL) architecture in DenseNet121.

A deeper look at FS and TL considering the metrics of loss and accuracy per epoch was presented in Figs. 2 and 3, respectively. In both architectures, loss (which is the combination of errors after iterations) decreases progressively with the epochs, while accuracy increases, both during training and validation setups. TL, however, shows a more evident reduction of loss over time—within a shallow curve that ends close to zero by the end of the 100 epochs. This phenomenon is not observed in FS. Additionally, the accuracy of TL is represented by a more curvilinear improvement that starts over 0.5 increasing to nearly 1. In FS, the accuracy curve starts over 0.6 (initially better) and stabilizes when it reaches 0.9. These outcomes show that TL had better improvement over sequential iterations.

**Figure 4:**
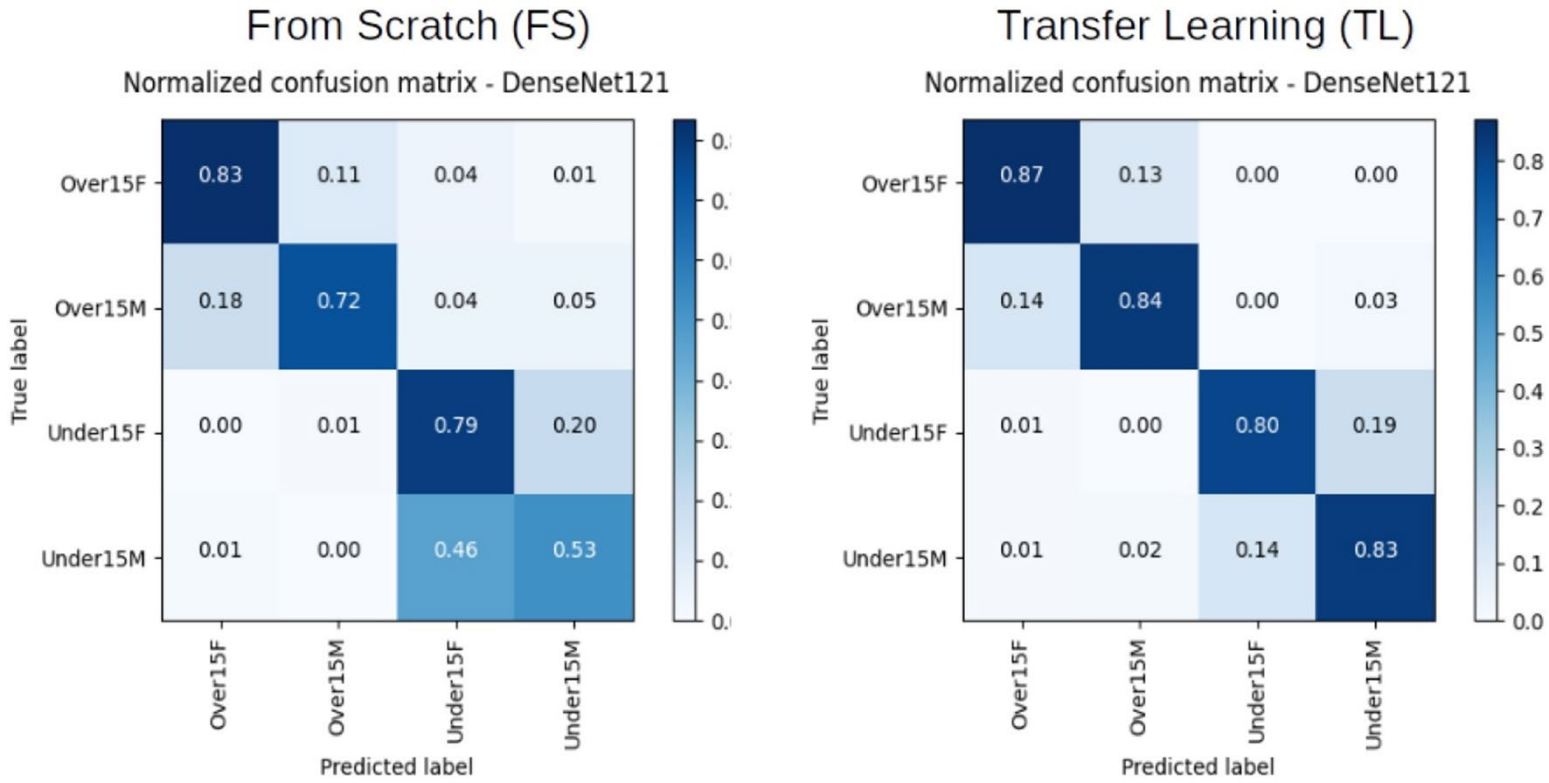
Normalized Confusion Matrix with the classification frequencies for each group set in the learning model. Outcomes presented for DenseNet121 using From Scratch (FS) and Transfer Learning (TL) architectures.

**Figure 5:**
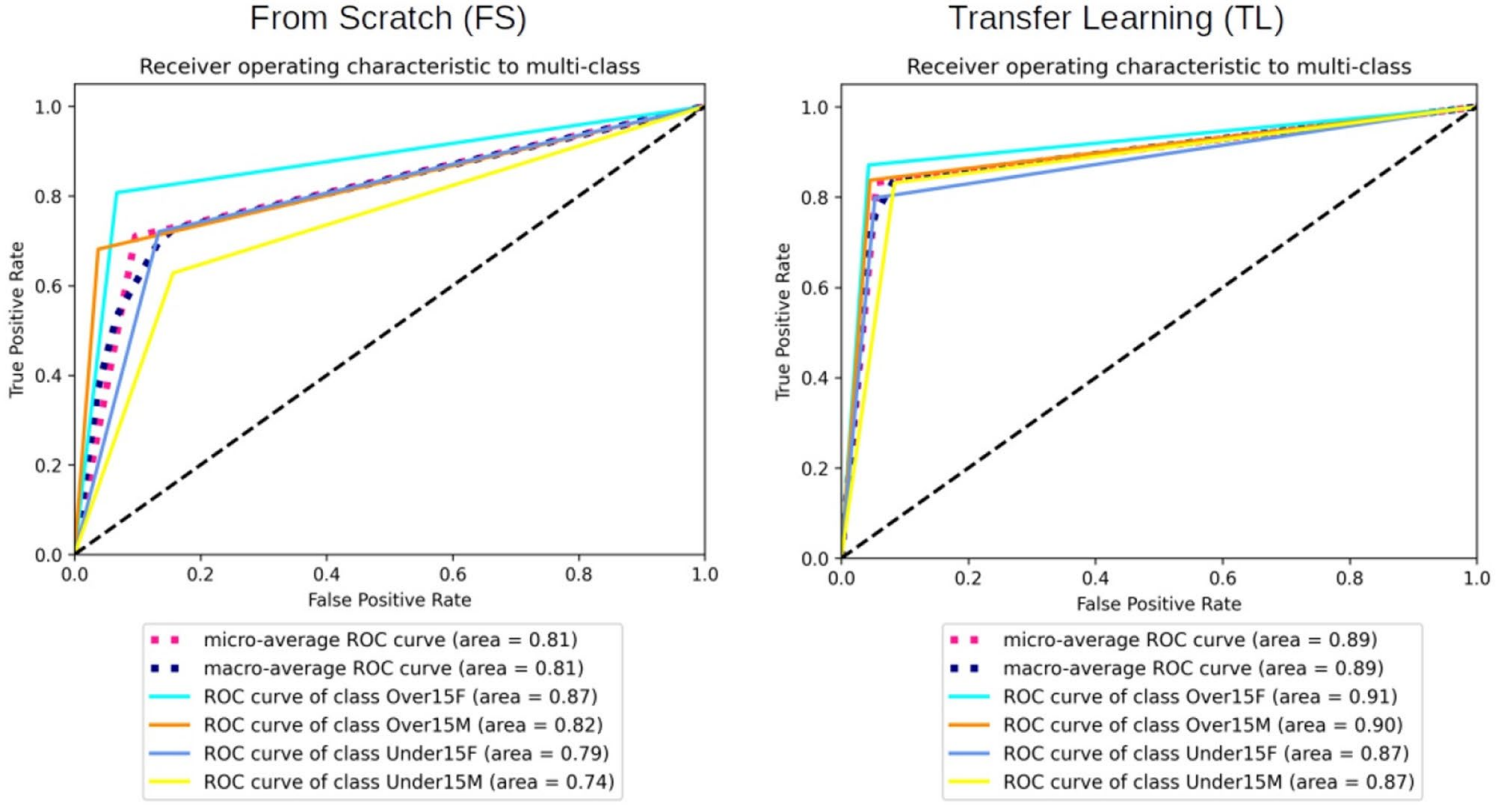
Receiver Operating Characteristic (ROC) curves to MultiClass analyses using DenseNet121 with From Scratch (FS) and Transfer Learning (TL) architectures.

Figure 4 shows the confusion matrix for the performance of DenseNet121 to classify males and females in the age groups below and above (or equal) 15 years. In the older group, FS approach reached 0.83 and 0.72 for the correct classification of females and males, respectively. In the younger group, the classification rates decreased to 0.79 and 0.53, respectively. With TL, the correct classification of females and males in the older group reached 0.87 and 0.84, respectively, while in the younger group the classification rates decreased to 0.80 and 0.83, respectively. The optimal performance of TL over FS within DenseNet121 is visualized in Fig. 5.

ROC curves for FS showed AUC of 0.87 and 0.82 for the classification of females and males above (or equal) the age of 15 years, and 0.79 and 0.74 for females and males below the age of 15 years. The AUC obtained with TL reached 0.91 and 0.90 for females and males in the younger age group, and 0.87 for both sexes in the younger age group.

**Figure 6:**
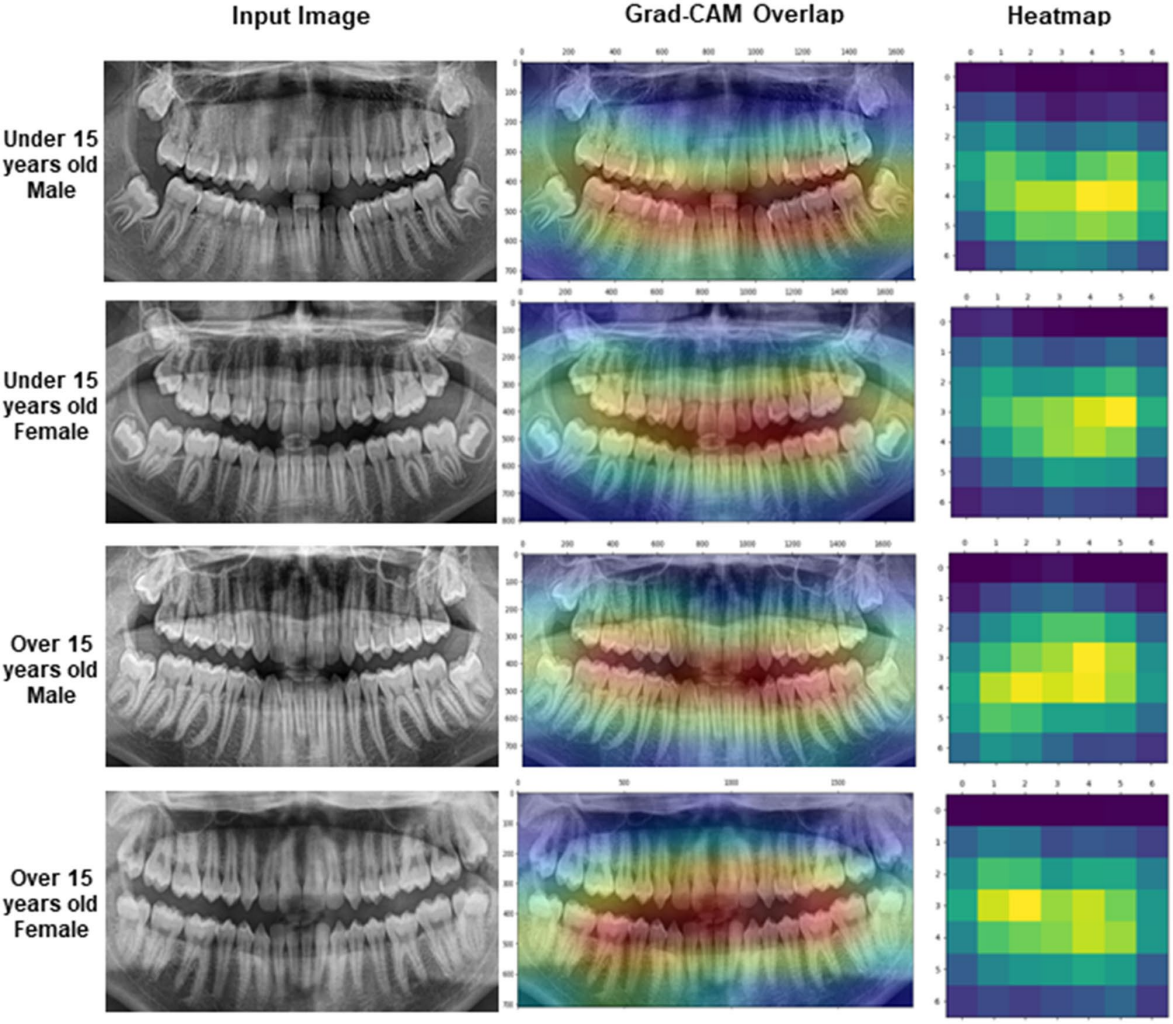
Samples of images representing the four classes used for the classification process with the representation of the Gradient-weighted Class Activation Mapping (Grad-CAM) and the scaled representation of the heatmap.

Finally, Fig. 6 shows the gradient-weighted class activation mapping (Grad-CAM) in which stronger signals (reddish) were observed around the crowns of anterior and posterior teeth. Weak signals (blueish) were observed in root and bone regions.

## Discussion

Sexual dimorphism is a crucial step in the anthropological process of building the biological profile of the deceased^37^. In general, sex-related differences between males and females are expressed as changes in the shape and size of anatomic structures^38^. Puberty is a biological landmark that triggers more evident differences between males and females^39^. Over time, these differences will manifest especially in the pelvic bones and the skull^40^. Teeth, however, are known for their resistance to environmental effects (extrinsic factors) and systemic health conditions (intrinsic factors); and are available for forensic examination in most cases. Moreover, the radiographic visualization of dental anatomy is optimal given the highly mineralized tissues of crown and root(s). This study proposed the use of artificial intelligence for the radio-diagnostic task of sexual dimorphism from human teeth.

A preliminary challenge proposed to test the artificial intelligence in this study was the inclusion of anatomically immature individuals in the sample. This is to say that the human skeleton is not fully influenced by the hormonal changes early in life and that the maxillofacial bones are still similar between males and females in childhood. More specifically, the age limits of the addressed population were 6 and 22.9 years—an interval that covers children, adolescents, and young adults. Deciduous and some permanent teeth, on the other hand, will express full development in childhood. The permanent mandibular first molar, for instance, shows apex closure around the age of 7.5 years. Aris et al.^39^, explain that teeth that fully develop long before puberty may have observable dimorphic features that can be explored even before the expression of skeletal dimorphism. Hence, the rationale at this point was to test the performance of the artificial intelligence within a scenario in which the mandible, maxillae, and other skulls bones would not play a major role in sexual dimorphism, giving the chance to teeth to express their dimorphic potential.

The radiographic aspect of the present study differs from the (physical) anthropological assessment of Aris et al.^39^, because our study has the preliminary and fundamental scope of screening teeth (or tooth regions) that can play a more important part to distinguish males and females. In a future step, teeth and tooth regions detected as dimorphic in the present study could be tested and validated by means of physical examination (i.e. studies ex vivo). Among the main advantages of the radiographic approach is the visualization of dental anatomy, including the internal aspect of the crown and roots (namely the pulp chamber and root canals, respectively), and the possibility of retrospective dataset sampling from existing databases—which is hampered in observational anthropological/archaeological studies.

DenseNet121 architecture running with TL training approach in 100 epochs led to the best performance for sexual dimorphism. Particularly, the training accuracy maintained high (above 80%) between epochs 19–100, while the validation accuracy was between 70–83% after epoch 31. Consequently, the average accuracy of TL was 82%, with average specificity of 92% in the total sample. Authors claim^41^ that when the entire skeleton is available for anthropological assessment, the accuracy of sexual dimorphism can reach 100%. This phenomenon is justified by the contribution of pelvic bones and skull to the analyses. Studies solely based on teeth present much lower estimates. Paknahad et al.^42^, for instance, performed a study with bitewing radiographs and reported an accuracy of 68% for sexual dimorphism based on odontometric assessments of the deciduous second molars (mandibular and maxillary). In our study, the higher accuracy rates are possibly justified by the integral assessment of dental anatomy (all the visible bidimensional dental features of the teeth were considered) in the process of sexual dimorphism—instead of specific linear measurements. In the study of Paknahad et al.^42^, only the width of the enamel, dentin, and pulp space were considered. Moreover, our study assessed radiographs of 4003 individuals, while the previous authors^42^ sampled only 124 individuals. In practice, a preliminary overall accuracy of 82% (specificity of 92%) corroborates DenseNet121 with TL approach as a proper tool for radiographic sexual dimorphism.

The purpose of the present study, however, was to challenge to artificial intelligence even more. To that end, the sample was divided into males and females below and above the age of 15 years. ROC curves obtained during the analyses per age category showed AUC between 0.90–0.91 for males and females over the age of 15, respectively, while in the younger group the AUC was 0.87 for both the males and females. These outcomes confirm that, in fact, sexual dimorphism is more challenging among children (in this case, between 6 and 14.9 years). In both groups, however, the AUC was considered excellent for diagnostic accuracy tests^43^. Consequently, the features assessed from panoramic radiographs in the present study had enough discriminant power to distinguish males and females with accurate performance.

The Grad-CAM images obtained in our study showed a similar region of activation in both age groups. In general, the activation region was more centralized and horizontal – surrounding the crowns of anterior and posterior teeth. These outcomes are corroborated by studies that show the dimorphic value of canines^44,45^ and incisors^41^ between males and females.

This is a preliminary study to understand the discriminant power of dental morphology to distinguish males and females using panoramic radiographs. At this point, these outcomes should not be translated to practice since they currently serve to screen regions of teeth that may weigh more for sexual dimorphism. A few cases in the scientific literature reported the use of postmortem panoramic radiographs for human identification^46,47^. In these cases, the current findings could have a more tangible application. For anthropological practices in single cases and mass disasters, more comprehensive knowledge of radiographic sexual dimorphism is needed, especially when it comes to the effects of age on dental morphological features.

## Conclusion

The dentomaxillofacial features assessed on panoramic radiographs in the present study showed discriminant power to distinguish males and females with excellent accuracy. Higher accuracy rates were observed among adolescents and young adults (older group) compared to children (younger group). DenseNet121 architecture with TL approach led to the best outcomes compared to FS. The regions with stronger activation signals for machine-guided sexual dimorphism were around the crowns of anterior and posterior teeth.

## Supplementary Information

Supplementary Information 1.

Supplementary Information 2.

Supplementary Information 3.

Supplementary Information 4.

## Data Availability

The datasets used and/or analysed during the current study available from the corresponding author on reasonable request.
