# Supplementary material for: Diagnostic performance of convolutional neural networks for dental sexual dimorphism
Source: Sci Rep. 2022 Oct 14;12:17279. doi: 10.1038/s41598-022-21294-1 (PMC9568558; doi:10.1038/s41598-022-21294-1)
Supplement: Supplementary file 1 — Supplementary Information 1. [file 41598_2022_21294_MOESM1_ESM.docx]

**Legends of Supplementary files**

**Suppl. I.** Loss and evolutionary accuracy of the training process and learning validation of all convolutional neural network (CNN) architectures tested in the preliminary step of this study. DenseNet121 presented the best performance among seven other CNNs.

**Suppl. II.** Confusion matrices of all the eight convolutional neural networks tested in the preliminary step of this study. The best classification performance was observed using DenseNet121.

**Suppl. III.** Receiver operating characteristic (ROC) curves obtained for the multi-class task performed with all the convolutional neural networks used in this study. The higher values for the area under the curve were obtained with DenseNet121.
