# Supplementary figures and images for: Diagnostic performance of convolutional neural networks for dental sexual dimorphism
Source: Sci Rep. 2022 Oct 14;12:17279. doi: 10.1038/s41598-022-21294-1 (PMC9568558; doi:10.1038/s41598-022-21294-1)

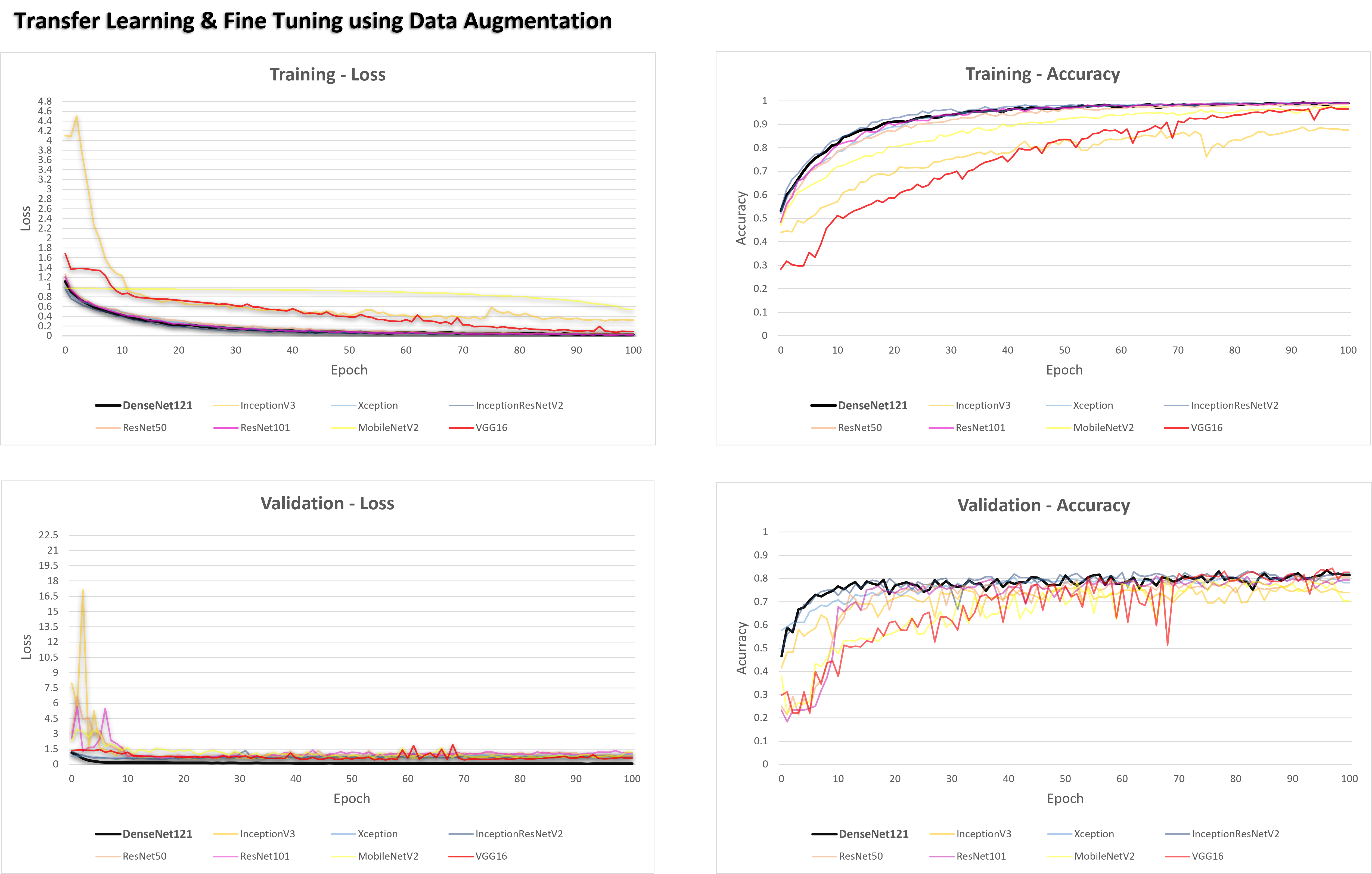

Supplement: Supplementary file 2 — Supplementary Information 2. [file 41598_2022_21294_MOESM2_ESM.png]

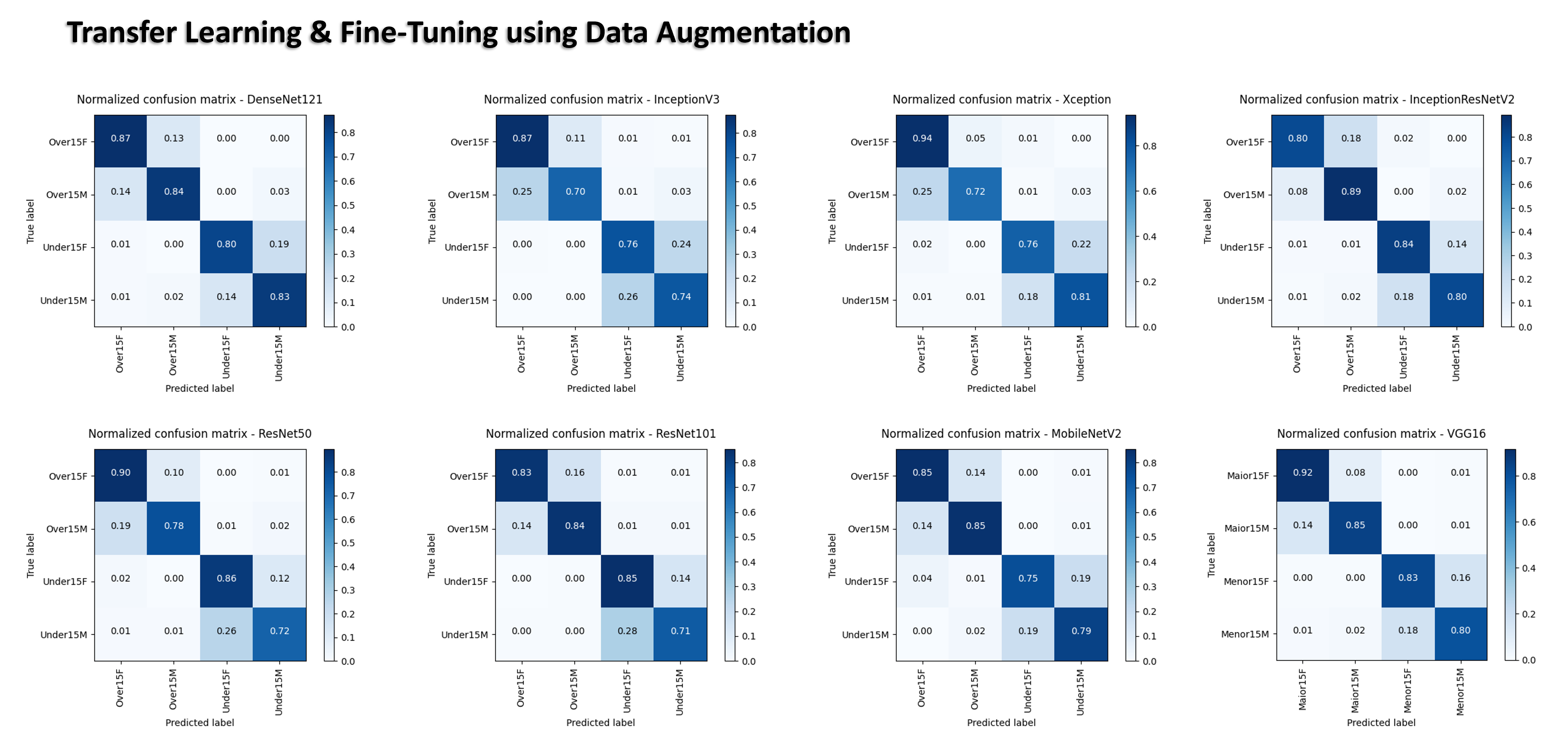

Supplement: Supplementary file 3 — Supplementary Information 3. [file 41598_2022_21294_MOESM3_ESM.png]

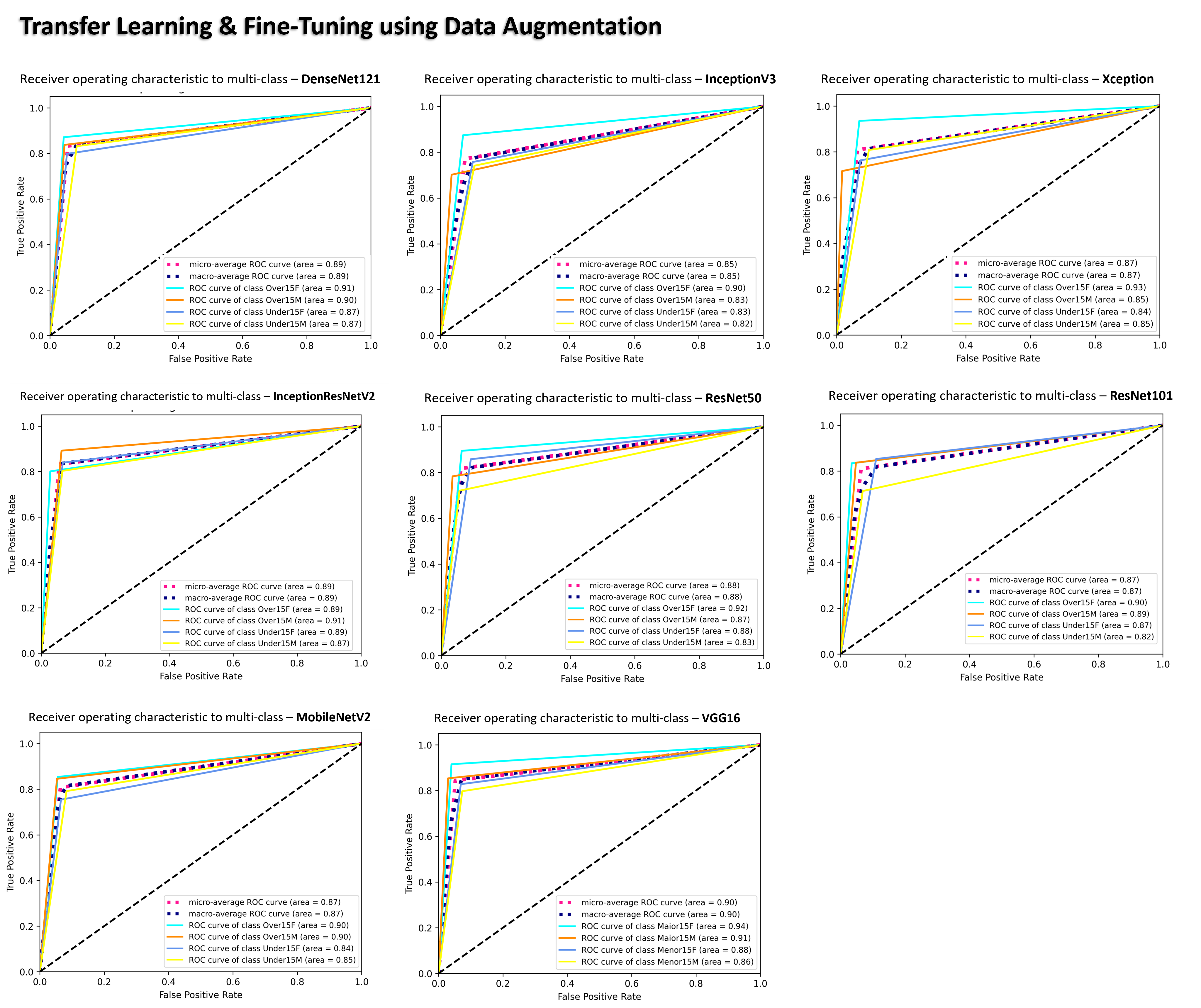

Supplement: Supplementary file 4 — Supplementary Information 4. [file 41598_2022_21294_MOESM4_ESM.png]
